# Monocular-Based 6-Degree of Freedom Pose Estimation Technology for Robotic Intelligent Grasping Systems

**DOI:** 10.3390/s17020334

**Published:** 2017-02-14

**Authors:** Tao Liu, Yin Guo, Shourui Yang, Shibin Yin, Jigui Zhu

**Affiliations:** State Key Laboratory of Precision Measuring Technology and Instruments, Tianjin University, Tianjin 300072, China; liutao1943@tju.edu.cn (T.L.); shouruiyang@tju.edu.cn (S.Y.); shibinyin@tju.edu.cn (S.Y.); jiguizhu@tju.edu.cn (J.Z.)

**Keywords:** monocular, industrial robot, intelligent grasping, pose estimation

## Abstract

Industrial robots are expected to undertake ever more advanced tasks in the modern manufacturing industry, such as intelligent grasping, in which robots should be capable of recognizing the position and orientation of a part before grasping it. In this paper, a monocular-based 6-degree of freedom (DOF) pose estimation technology to enable robots to grasp large-size parts at informal poses is proposed. A camera was mounted on the robot end-flange and oriented to measure several featured points on the part before the robot moved to grasp it. In order to estimate the part pose, a nonlinear optimization model based on the camera object space collinearity error in different poses is established, and the initial iteration value is estimated with the differential transformation. Measuring poses of the camera are optimized based on uncertainty analysis. Also, the principle of the robotic intelligent grasping system was developed, with which the robot could adjust its pose to grasp the part. In experimental tests, the part poses estimated with the method described in this paper were compared with those produced by a laser tracker, and results show the RMS angle and position error are about 0.0228° and 0.4603 mm. Robotic intelligent grasping tests were also successfully performed in the experiments.

## 1. Introduction

With the rapid development of the robotic manufacturing industry and the continuous decrease of robots’ production cost, industrial robots are being extensively used as economical and flexible orienting devices in modern industries [1,2]. According to the statistics, about 80% of the industrial robots are used in the automobile manufacturing industry where they do all kinds of jobs, including spot welding, picking and placing, drilling and cutting and so on. With the improvement of robot performance, the application level of the industrial robots has been raised higher and higher, and nowadays industrial robots are expected to undertake even more advanced tasks such as precision machining, high-accuracy autonomous inspection and intelligent control [3,4]. Also, the Industrie 4.0 [5] and the European Commission Horizon 2020 Framework Programme [6] have clearly pointed out the intelligent application direction of industrial robots. 

One of the most typical robot intelligent applications is robotic intelligent grasping in automotive manufacturing. During the car assembly process, different components and parts are installed in an orderly way at different workstations of the production line, and eventually assembled into a complete car. In the conventional automobile workshops, a couple of employees are arranged at each workstation, and they are responsible for taking parts out of the work bins, and placing them onto a high-accuracy centering device. Then, the robots grasp the parts from the centering device, and install them onto the car. Industrial robots are quite competent at these jobs due to their high repeatability. However, as problems of labor-force shortages, high labor costs, harsh working environments and unstable personnel operation become increasingly prominent, manufacturing enterprises have been eagerly awaiting the time when robots can grasp parts from the work bins directly instead of by manual operation, so robotic intelligent grasping has inevitably risen to be an trend in modern automobile manufacturing.

In automobile manufacturing, car body parts are placed in different ways, for example, roofs are often stacked in the work bins, doors are usually placed on shelves, side-walls are hung on an electric monorail system (EMS), and some smaller parts are placed directly on the conveyor belt. In these circumstances, the position and orientation of these parts are uncertain, and may have a ±50 mm position deviation or a ±5° angle offset in some extreme cases. Generally, the accuracy requirement for operations such as grasping will be of the order of 0.5 mm. In order to grasp these imprecisely-placed parts, the robot should adjust its trajectory according to the pose of the part and align its gripper to it. In other words, the robot should be able to recognize the pose of a part before grasping it. 

There are some real-time position and orientation measurement devices that can be used for robot pose tracking and guiding, such as the indoor global positioning system (iGPS) [7,8], and the workspace measuring and positioning system (wMPS) [9]. However, in this equipment, the position of a spatial point is measured by ensuring that the optical information from two or more measuring stations is acquired, but this requirement cannot be satisfied in most cases because of the occlusion caused by many factors, including the complex structure of the moving objects, obstacles and physical obstructions in the working volume, and the limited field of view of the optical devices.

In recent years, vision measurement technology, which features non-contact, high accuracy, real-time and the on-line measurement, has developed fast and become used widely [10]. The robotic visual inspection system, which combines vision measurement technology and robot technology has been applied extensively in the automotive and aircraft manufacturing industries [11,12,13]. Therefore determining the part pose with vision measurement technology before the robot grasps the part may be a feasible means to realize intelligent robot grasping. There are several different robotic vision solutions such as monocular vision [14], binocular vision [15], structured light method [16], and the multiple-sensor method [17], that have proven their suitability in the robotic visual inspection tasks, but in general we cannot get three dimensional (3D) information about parts with a single camera [18]. The structured light method needs laser assistance and the measuring range is limited by the laser triangular measurement principle [19]. Binocular vision systems have a complex configuration that needs the relative transform relationship calibration (also called extrinsic parameters calibration) and time-consuming algorithms for feature extraction and stereo matching, and they are also not suitable for parts with complex shapes and large surface curvature changes [20,21,22]. As most automobile parts are molded by the punch forming method, featured points on the parts have great consistency and small dimensional deviations. We could develop a low-cost and high-adaptability 6D pose (3-DOF of translations and 3-DOF of angles) estimation method by combining the geometric information of parts and visual measurement technology [23,24]. 

In this paper, we will introduce a monocular-based 6D pose estimation method with the assistance of the part geometry information, which aims at enabling industrial robots to grasp automobile parts, especially those with large-sizes, at informal poses. An industrial camera is mounted on the end of the robot with the gripper, and the robot uses the camera to measure several featured points on the part at different poses. Due to the robot’s high repeatability but low accuracy, the measuring pose of the camera in each robot position will be consistent and can be calibrated by high-accuracy measuring equipment such as a laser tracker. The positions of the featured points on the part can be also calibrated beforehand. A nonlinear optimization model for the camera object space collinearity error in different poses is established, and the initial iteration value is estimated based on differential transformation and the 6-DOF pose of the part is thus determined with more than four featured points. In addition, based on the uncertainty analysis theory, the measuring poses of the camera are optimized to improve the estimation accuracy. Finally, based on the principle of the robotic intelligent grasping system developed in this paper, the robot could adjust its learned path and grasp the part.

The rest of the paper is organized as follows: Section 2 introduces the principle of the robot intelligent grasping system and the system calibration method. The mathematic model of our monocular 6-DOF pose estimation technology is presented in Section 3. Then the uncertainty analysis and camera pose optimization method are given in Section 4. Experimental tests and results are given in Section 5. The paper will finish in Section 6 with a short conclusion and a summary.

## 2. Robotic Intelligent Grasping Systems

### 2.1. Principle of the Robotic Intelligent Grasping System

In a robotic loading workstation, the robot grasps components or parts from work bins or shelves and then assembles them onto the car body. Components and parts vary in size and differ in shape, as small-sized parts such as a top rail or a wheel fender may be less than 0.5 m × 0.5 m, while large-sized parts such as a roof or a side-wall may be larger than 2 m × 1 m. As there are no precise positioning devices for work bins or shelves, i.e., the poses of the parts are usually uncertain, the position deviation between different parts can be as large as ±50 mm. In order to determine the pose of each part and guide the robot to grasp it precisely, a camera is mounted on the robot end-flange to measure the pose of the part, then the robot will adjust the pose of the gripper according to the measured part pose.

**Figure 1:**
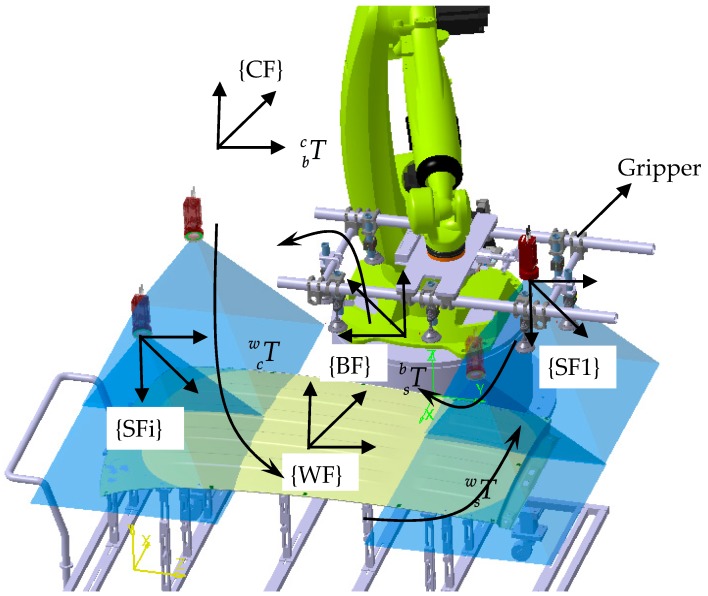
Schematic of robot intelligent grasping system.

As shown in Figure 1, before the robot moves to grasp a part, it moves and locates the camera to measure several featured points on it, and then determines the part pose based on the measurement model. In order to enlarge the camera’s field of view, a short focal length lens was mounted on it. For small-size parts, the featured points could be included in one field of view of the camera, and the robot only needs to move to one position, but for a large-size part, the featured points will have a wider distribution, and the robot needs to move to several positions. As the former is a special case of the latter, the large-size part is discussed in this paper. The coordinate systems of the robotic intelligent grasping system consist of cell frame (CF), robot base frame (BF), part frame (WF) and camera pose frame (SF). As the camera was located to several poses in order to measure the featured points on the large-size parts, a SFi is defined at each measuring pose. 

The 1-st camera frame (SF1) was taken as the reference frame in this method, that is pose of the part was determined relative to SF1. The part frame (WF) is defined based on the CAD model or established with several featured points on it. The principle of the robotic intelligent grasping system is described in detail as follows:

(1) A robot measuring path was taught, along which the robot could take the camera to measure the featured points on the large-size part, and the part pose relative to SF1 (Tws) could be determined.
(2) For an initial part, an initial grasping path was taught, along which the robot could grasp the initial part precisely. And pose of the initial part relative to SF1 (Tw0s) was measured and recorded. As shown in Figure 1, the part pose relative to SF1 can be given as follows:
  (1) Tw0s−1=Tsw0=Tcw0×Tbc×Tsb
  where Tsb is the transform relationship between SF1 and BF; Tbc is the transformation between BF and CF; and Tcw0 is the transformation between CF and the initial part frame (WF). Then, for a practical part that has a pose deviation with the initial part, the robot takes the camera to measure the part along the measuring trajectory, and determines the pose of the part relative to SF1 (Twis), which can be given as follows:
  (2) Twis−1=Tswi=Tcwi×Tbc×Tsb
  As the industrial robot is fixed in the station, transform relationship between BF and CF (Tbc) is invariable. Also as the camera system adopted in this method has a wide field of view, it can include the featured points on the practical deviated parts at the same robot pose. Because of the high repeatability of the industrial robot, the pose of SF1 relative to BF (Tsb) can be regard as fixed.
(3) Finally, in order to grasp a practical part, the robot pose should be adjusted so that the pose of the gripper could align with the part pose, that is, the pose of the practical part relative to the gripper should be the same as the initial part. As the camera is mounted on the robot end-flange, the relative position between the camera and the gripper is fixed, the robot pose adjustment can be also regard as aligning the camera pose with the part pose. After the robot pose adjustment, the pose of the practical part relative to SF1 is the same as the initial part, that is:
  (3) Tsw0=Tcwi×Tbc×Tsbi
  where Tsbi is the transform relationship between the adjusted SF1 and BF.

In the robot control system, the pose adjustment can be realized by base frame offset, changing the transform relationship between the BF and the CF with an offset transformation matrix:

(4) Tbc×Tsbi=ΔTbc×Tbc×Tsb

where ΔTbc is the base frame offset transformation matrix.

Combining Equations (2)–(4), we can obtain the base frame offset transformation matrix as:

(5) ΔTbc=Tswi−1×Tsw0

As Tsw0 and Tswi are the determined pose of the initial part and the practical part relative to SF1, we could calculate the base frame offset transformation matrix ΔTbc, with which the robot could adjust its pose based on the coordinate transformation principle and robot inverse kinematics. The trajectory adjustment was performed in the robot controller. The base frame offset is also acting on the taught grasping path, and the robot could move to grasp the practical part along the adjusted grasping path, thus achieving intelligent grasping.

### 2.2. System Calibration Method

The calibration of the robotic intelligent grapping system is to determine the transform relationship between different frames, including the cell frame (CF), robot base frame (BF), part frame (WF) and *i*-th camera pose frame (SFi). In this paper, a laser tracker is used as the measuring equipment, and CF is set up by measuring several benchmarks on the workstation. Although CF is not necessary, it is usually defined in the practical workshop to relate different robots, so we keep it in this paper.

As BF is defined based on the robot’s mechanical structure, it cannot be measured directly. In this paper, a sophisticated tool is mounted on the robot flange to hold a 1.5 inch spherically mounted reflector (SMR) of a laser tracker, and the center of the SMR is defined as the tool center point (TCP). The robot is controlled to move to several different positions with different postures, and the positions of the SMR are measured by the laser tracker. Then the transformation between the BF and the laser tracker is calculated based on the measured SMR positions and robot kinematics.

The part calibration process includes measuring the coordinates of the featured points in WF and calibrating the transform relationship between WF and CF. An initial pose is defined for the initial part, the laser tracker measures several featured points on the part and WF can be established based on these points. As CF has been measured and established before, the transform relationship between WF and CF can be calculated based on the principle of rigid body transformation.

**Figure 2:**
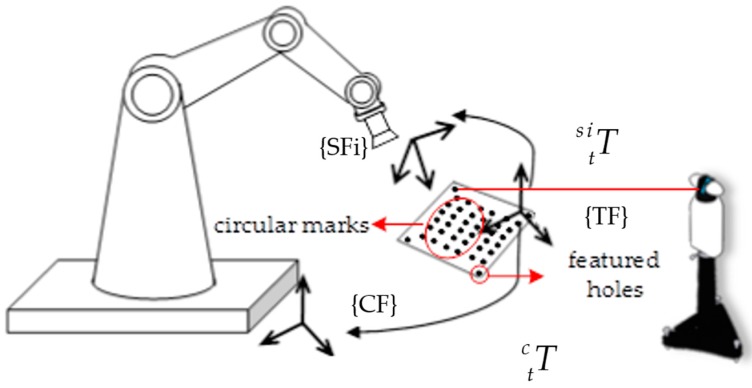
Calibration of the camera *i*-th pose frame.

After the robot’s measuring trajectory has been taught, pose of the camera measuring pose frames (SFi) need to be calibrated. As coordinate system of a camera is defined based on its optical structure, which cannot be measured with the laser tracker directly. In this paper, the camera pose frames were calibrated based on homography and principle of rigid body transformation, which was illustrated in Figure 2. A calibration target with circular marks and several featured holes is adopted, the target frame (TF) is defined by the featured holes and the circular marks have been calibrated in the target frame. When the camera stops at a measuring pose, it captures image of the calibration target, the transform relationship between SFi and the target frame can be calculated with the homography, and the target frame can be measured by the laser tracker. Then the transform relationship between SFi and CF can be calculated as follows:

(6) Tsic=Ttc×(Ttsi)−1

## 3. The Mathematic Models for Monocular 6-DOF Pose Estimation Technology

The monocular-based 6-DOF pose estimation technology is mainly based on the camera pinhole model and the principle of rigid body transformation. Unlike similar pose estimation techniques with a single camera, the featured points in this method are low in number and wide in distribution, the camera is mounted on the robot end-flange and the robot has to move to several different poses to capture pictures of these featured points. The principle of 6-DOF pose estimation technology based on a single camera and couple of wide-distributed featured points is discussed in detail here.

### 3.1. Pinhole Camera Model

**Figure 3:**
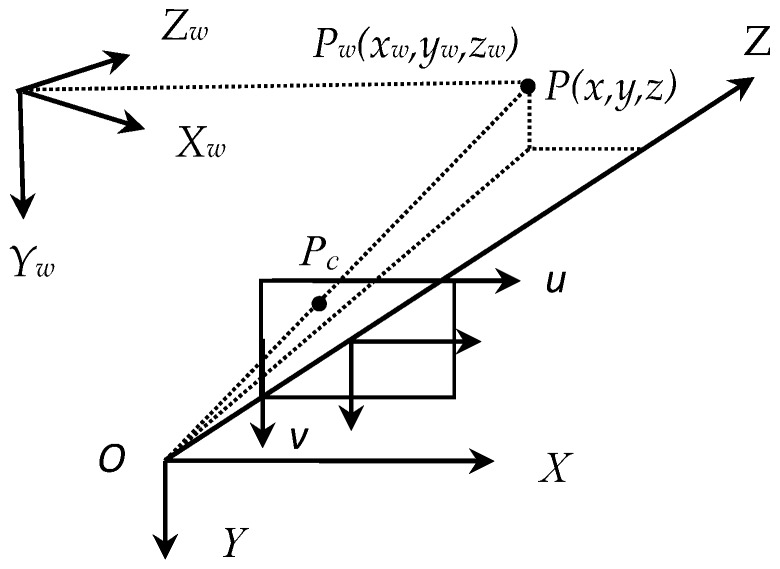
Schematic of the pinhole camera model.

As shown in Figure 3, the ideal pinhole camera model is used to represent the relationship between the object space and the image space, the relationship between the coordinate of the object point *P_w_* and the image point *P_c_* is given as follows:

(7) $$zP_{c}=A\times T\times P_{w}$$

where *z* is the third row of *T* × *P_w_*, and *P_w_* = [*x_w_ y_w_ z_w_* 1]*^T^* is the coordinate of a spatial point in the object space and *P_c_* = [*u v* 1]*^T^* is its corresponding coordinate in image space. $T=\left[\begin{array}{cc} R & t\\ 0 & 1 \end{array}\right]$ is the camera extrinsic parameter, which represent the relationship between the camera coordinate system and the object coordinate system, and $A=\left[\begin{array}{cccc} a_{x} & \gamma & u_{0} & 0\\ 0 & a_{y} & v_{0} & 0\\ 0 & 0 & 1 & 0 \end{array}\right]$ is the camera intrinsic matrix which represent the relationship between the camera coordinate system and its image space. *a_x_* and *a_y_* are the effective focal length in pixels of the camera along the *x* and *y* direction, (*u*_0_, *v*_0_) is the coordinate of the principle point, *γ* is the skew factor which is usually set to zero. 

The ideal pinhole camera model has not considered the distortion of camera lens, but for a real imaging system, lens distortion in the radial and tangential directions is unavoidable due to mechanical imperfection and lens misalignments. Considering the impact of lens distortion, the relationship between the ideal image point [*u* *v*]*^T^* and the distorted point [*u’* *v’*]*^T^* is:

(8) $$\left[\begin{array}{c} x_{p}\\ y_{p} \end{array}\right]=\left(1+k_{1}r^{2}+k_{2}r^{4}\right)\left[\begin{array}{c} x_{d}\\ y_{d} \end{array}\right]+\left[\begin{array}{c} 2p_{1}x_{d}y_{d}+p_{2}\left(r^{2}+2x_{d}{}^{2}\right)\\ p_{1}\left(r^{2}+2y_{d}{}^{2}\right)+2p_{2}x_{d}y_{d} \end{array}\right]$$

where *x_p_* = (*u’ − u*_0_)/*a_x_*, *y_p_* = (*v’ − v*_0_)/*a_y_*, *x_d_* = (*u − u*_0_)/*a_x_*, *y_d_* = (*v − v*_0_)/*a_y_*, $r^{2}=x_{d}^{2}+y_{d}^{2},$ [*k*_1_ *k*_2_ *p*_1_ *p*_2_] are the lens distortion coefficients. The coordinates of image point *P_c_* in the following paper are the pixel coordinates after distortion correction.

### 3.2. Camera Multi-Pose Measurement Model

Determining the rigid transformation that relates images to the known geometry, pose estimation problem, is one of the central problems in photogrammetry, robotics vision, computer graphics, and computer vision. However, the pose estimation problem discussed in this paper is different from the common ones: the camera needs to take pictures of widely-distributed featured points at multiple poses instead of only one pose. The most significant advantage of the method presented in this paper is that it can be adopted for pose estimation of large-size part, much larger than the camera’s field of view. The measurement model is presented in detail as follows. 

In order to relate the measured results of different featured points, the coordinate transform relationship among different camera measuring poses should be calibrated in advance. Taking the first camera pose (SF1) as reference, the pinhole model of camera at the *i*-th measuring pose is:

(9) zPci=A×Twsi×Pwi=A×Ts1si×Tws1×Pwi

where *P_ci_* = [*u_i_ v_i_* 1]*^T^* is the pixel image coordinate of a featured point, *P_wi_* = [*x_wi_ y_wi_ z_wi_* 1]*^T^* is its corresponding coordinate in the part frame, which is calibrated with the method in Section 2.2. Ts1si is the transform relationship between the first camera pose (SF1) and the *i*-th camera pose (SFi), which is also calibrated with the method presented in Section 2.2. Tws1=[r11r12r13t1r21r22r23t2r31r32r33t30001] is the relationship between SF1 and the WF, which is the part pose to be estimated in this method.

The image point *P_ci_* is usually normalized by the internal parameter matrix *A* of the camera to obtain the normalized image point, and let ${\tilde{m}}_{i}$ be the direction vector of the line linking the principle point and the image point:

(10) $${\tilde{m}}_{i}=A^{-1}\times P_{ci}$$

where $A=\left[\begin{array}{ccc} a_{x} & 0 & u_{0}\\ 0 & a_{y} & v_{0}\\ 0 & 0 & 1 \end{array}\right]$.

Let:

(11) Mi=(Ts1si×Tws1×Pwi)(1:3)=[xyz]T

The normalized image point is the projection of *M_i_* on the normalized image plane. According to the collinearity equation, *M_i_*, the normalized image point and principle point should be collinear under the idealized pinhole camera model, which can be expressed as follows:

(12) $${\tilde{m}}_{i}=\frac{M_{i}}{z}$$

Another way of considering the collinearity property is the orthogonal projection of the spacial point *M_i_* on the direction vector ${\tilde{m}}_{i}$ should be *M_i_* itself, that is:

(13) $$M_{i}=U_{i}\times M_{i}$$

where $U_{i}=\frac{{\tilde{m}}_{i}\times {\tilde{m}}_{i}{}^{T}}{{\tilde{m}}_{i}{}^{T}\times {\tilde{m}}_{i}}$ is the line-of-sight projection matrix which could project a scene point orthogonally to the line of sight constructed by the image point and the principle point.

**Figure 4:**
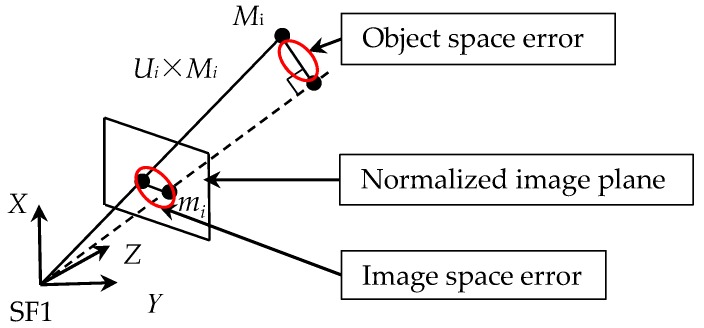
Image space error and the object space error.

As shown in Figure 4, the collinearity error can be considered in the image space or in the object space, in this paper we consider to penalize the collinearity error in the object space:

(14) $$F_{i}=\left(I-U_{i}\right)\times M_{i}=0$$

Although Equation (14) can be decomposed into three linear equations, the rank of its coefficient matrix is 2, that means we can only obtain two independent equations for a featured point. During the practical measurement, with n featured points on the part, we can formulate a function system with 2n equations about the elements of the matrix Tws1.

Because of the coefficient error in Equation (14), which is caused by the measurement error, the rotation matrix of Tws1 does not in general satisfy the orthogonal constraint, so in this paper, we adopt a nonlinear method to determine Tws1, which will consider the orthogonal constraint condition as follows:

(15) $$\left\{ \begin{array}{l} f_{1}=r_{11}{}^{2}+r_{21}{}^{2}+r_{31}{}^{2}-1=0\\ f_{2}=r_{12}{}^{2}+r_{22}{}^{2}+r_{32}{}^{2}-1=0\\ f_{3}=r_{13}{}^{2}+r_{23}{}^{2}+r_{33}{}^{2}-1=0\\ f_{4}=r_{11}\times r_{12}+r_{21}\times r_{22}+r_{31}\times r_{32}=0\\ f_{5}=r_{12}\times r_{13}+r_{22}\times r_{23}+r_{32}\times r_{33}=0\\ f_{6}=r_{13}\times r_{11}+r_{23}\times r_{21}+r_{33}\times r_{31}=0 \end{array}\right.$$

In principle, the unknown Tws1 can be determined by the solution of the equations provided by only three featured points, in consideration of the measurement error and efficiency, we chose at least four featured points to improve the accuracy.

Based on Equations (14) and (15), Tws1 can be obtained by minimizing the following function:

(16) $$E=\sum_{i=1}^{n}\left(F_{i1}{}^{2}+F_{i2}{}^{2}\right)+M\sum_{i=1}^{6}f_{i}{}^{2}=\min$$

where M is the penalty factor. Levenberg-Marquardt algorithm [25,26,27] is used to solve the nonlinear minimization problem in Equation (15).

### 3.3. Estimation of the Initial Iteration Value

For most iterative optimization algorithms, a relatively accurate estimation of the initial iteration value is generally necessary. This is not only for an accurate result, but also to reduce the number of iterations. Consequently, we adopt an estimation method to obtain a relatively accurate initial value. The method is expounded as follows.

In the application of the robotic intelligent grasping, deviation between poses of the initial part and the practical part is relatively small comparing with the part size. So the pose of the initial part and the practical part relative to SF1 can be associated based on the differential transformation:

(17) Tws1=Tw0s1+Tw0s1×δT=Tw0s1×(I+δT)

where $\delta T=\left[\begin{array}{cccc} 0 & -\delta z & \delta y & dx\\ \delta z & 0 & -\delta x & dy\\ -\delta y & \delta x & 0 & dz\\ 0 & 0 & 0 & 0 \end{array}\right]$ is the differential transformation matrix. The differential transformation is based on Taylor expansion and is usually used to deal with non-linear problems. 

Then Equation (11) can be rewritten as:

(18) Mi=[Ts1si×Tw0s1×(I+δT)×Pwi](1:3)=[xyz]T

and Equation (14) can be expanded as:

(19) $$\begin{array}{l} \left(U_{i}-I\right)\times M_{i}=\left[\begin{array}{ccc} u_{11i}-1 & u_{12i} & u_{13i}\\ u_{21i} & u_{22i}-1 & u_{23i}\\ u_{31i} & u_{32i} & u_{33i}-1 \end{array}\right]\left[\begin{array}{cccc} T_{11} & T_{12} & T_{13} & T_{14}\\ T_{21} & T_{22} & T_{23} & T_{24}\\ T_{31} & T_{32} & T_{33} & T_{34} \end{array}\right]\left[\begin{array}{c} x_{wi}\\ y_{wi}\\ z_{wi}\\ 1 \end{array}\right]+\\ \left[\begin{array}{ccc} u_{11i}-1 & u_{12i} & u_{13i}\\ u_{21i} & u_{22i}-1 & u_{23i}\\ u_{31i} & u_{32i} & u_{33i}-1 \end{array}\right]\left[\begin{array}{cccc} T_{11} & T_{12} & T_{13} & T_{14}\\ T_{21} & T_{22} & T_{23} & T_{24}\\ T_{31} & T_{32} & T_{33} & T_{34} \end{array}\right]\left[\begin{array}{cccc} 0 & -\delta z & \delta y & dx\\ \delta z & 0 & -\delta x & dy\\ -\delta y & \delta x & 0 & dz\\ 0 & 0 & 0 & 0 \end{array}\right]\left[\begin{array}{c} x_{wi}\\ y_{wi}\\ z_{wi}\\ 1 \end{array}\right]=0 \end{array}$$

where $U_{i}=\left[\begin{array}{ccc} u_{11i} & u_{12i} & u_{13i}\\ u_{21i} & u_{22i} & u_{23i}\\ u_{31i} & u_{32i} & u_{33i} \end{array}\right]$is the line-of-sight projection matrix; $P_{wi}={\left[\begin{array}{cccc} x_{wi} & y_{wi} & z_{wi} & 1 \end{array}\right]}^{T}$ is the corresponding coordinate in WF; Ts1si×Tw0s1=[T11T12T13T14T21T22T23T24T31T32T33T340001] is a transformation matrix of the initial part, which can be also calibrated beforehand. Equation (19) can be rewritten as:

(20) $$\begin{array}{l} \left[\begin{array}{ccc} u_{11i}-1 & u_{12i} & u_{13i}\\ u_{21i} & u_{22i}-1 & u_{23i}\\ u_{31i} & u_{32i} & u_{33i}-1 \end{array}\right]\left[\begin{array}{ccc} T_{11} & T_{12} & T_{13}\\ T_{21} & T_{22} & T_{23}\\ T_{31} & T_{32} & T_{33} \end{array}\right]\left[\begin{array}{cccccc} 0 & -z_{wi} & y_{wi} & -1 & 0 & 0\\ z_{wi} & 0 & -x_{wi} & 0 & -1 & 0\\ -y_{wi} & x_{wi} & 0 & 0 & 0 & -1 \end{array}\right]\left[\begin{array}{c} \delta x\\ \delta y\\ \delta z\\ dx\\ dy\\ dz \end{array}\right]=\\ \left[\begin{array}{ccc} u_{11i}-1 & u_{12i} & u_{13i}\\ u_{21i} & u_{22i}-1 & u_{23i}\\ u_{31i} & u_{32i} & u_{33i}-1 \end{array}\right]\left[\begin{array}{cccc} T_{11} & T_{12} & T_{13} & T_{14}\\ T_{21} & T_{22} & T_{23} & T_{24}\\ T_{31} & T_{32} & T_{33} & T_{34} \end{array}\right]\left[\begin{array}{c} x_{wi}\\ y_{wi}\\ z_{wi}\\ 1 \end{array}\right] \end{array}$$

Also, the rank of the coefficient matrix in Equation (20) is 2, so we can only acquire two equations for one featured point *P_wi_*. With n featured points on the part, we can formulate a function system with 2n linear equations, and then a matrix equation can be obtained in form of *Ax* = *B * with *x* = [δ*x* δ*y* δ*z dx dy dz*]*^T^*. *x* can be solved by means of the least-squares method as follows:

(21) $$x={\left(A^{T}\times A\right)}^{-1}A^{T}\times B$$

Note that the Equation (17) is based on the approximation condition that the rotation angle θ between the practical part and the initial part should be small and we make the following assumption:

(22) $$\left\{ \begin{array}{l} \underset{\theta \to 0}{\lim}\sin\theta \to 0\\ \underset{\theta \to 0}{\lim}\cos\theta \to 1 \end{array}\right.$$

The approximation condition in Equation (22) means that the deviation between the pose of the practical part and the pose of the initial part should be small. When the rotation angle is large, the differential transformation will introduce significant error, so that solution for Equation (20) can only offer an estimation of the initial iteration value for Equation (15).

## 4. Camera Pose Optimization

As described above, the camera was mounted on the robot end-flange and oriented to measure the featured points on the part from different robot pose. Theoretically, the measuring poses of the camera were significant to the final accuracy of the part pose estimation. In order to optimize the camera poses and obtain higher accuracy, the uncertainty transitive model of the objective function was analyzed here.

Mathematically, the model for a multivariate system takes the form of an implicit relationship:

(23) Φ(X,P)=0

where *P* is measured or influence quantities (the input quantities), *X* is the measurand (the output quantities).

For the system described in this paper, *X* is the parameters of the transform relationship between SF1 and WF (Tws1) and *P* is the image coordinates of the featured points extracted from the captured images.

(24) $$\left\{ \begin{array}{l} X={\left[\begin{array}{cccccccccccc} r_{11} & r_{12} & r_{13} & t_{1} & r_{21} & r_{22} & r_{23} & t_{2} & r_{31} & r_{32} & r_{33} & t_{3} \end{array}\right]}^{T}\\ P={\left[\begin{array}{ccccccc} u_{1} & v_{1} & u_{2} & v_{2} & \cdots & u_{n} & v_{n} \end{array}\right]}^{T} \end{array}\right.$$

and Equation (16) can be rewritten as:

(25) $$E\left(X,P\right)=\sum_{i=1}^{n}\left(F_{i1}{}^{2}+F_{i2}{}^{2}\right)+M\sum_{i=1}^{6}f_{i}{}^{2}=\min$$

In order to meet the minimization condition *E*(*X,P*) = min, the derivative of *X* in Equation (25) should equal to zero, that is:

(26) $$\Phi \left(X,P\right)=\frac{\partial E\left(X,P\right)}{\partial X}=\left[\begin{array}{c} \frac{\partial E\left(X,P\right)}{\partial r_{11}}\\ \frac{\partial E\left(X,P\right)}{\partial r_{12}}\\ \vdots \\ \frac{\partial E\left(X,P\right)}{\partial t_{3}} \end{array}\right]=0$$

In this paper, covariance matrix *Q_X_* and *Q_p_* are used to express the measuring uncertainty of *X* and *P* as follows:

(27) $$\begin{array}{l} Q_{X}=diag\left\{\begin{array}{cccc} \sigma_{r_{11}}{}^{2} & \sigma_{r_{12}}{}^{2} & \cdots & \sigma_{t_{3}}{}^{2} \end{array}\right\}\\ Q_{p}=diag\left\{\begin{array}{ccccccc} \sigma_{u_{1}}{}^{2} & \sigma_{v_{1}}{}^{2} & \sigma_{u_{2}}{}^{2} & \sigma_{v_{2}}{}^{2} & \cdots & \sigma_{u_{n}}{}^{2} & \sigma_{vn}{}^{2} \end{array}\right\} \end{array}$$

where $\sigma_{u_{i}}{}^{2}$and $\sigma_{v_{i}}{}^{2}$ are the uncertainties of the extracted pixel coordinates of the featured points in image *x* and *y* direction.

Based on the derivation method of implicit function, *Q_p_* and *Q_X_* satisfy the following relationship:

(28) $$\left(\frac{\partial \Phi \left(X,P\right)}{\partial X}\right)Q_{X}{\left(\frac{\partial \Phi \left(X,P\right)}{\partial X}\right)}^{T}=\left(\frac{\partial \Phi \left(X,P\right)}{\partial P}\right)Q_{p}{\left(\frac{\partial \Phi \left(X,P\right)}{\partial P}\right)}^{T}$$

and *Q_X_* can be expressed as follows:

(29) $$Q_{X}=HQ_{P}H^{T}$$

with $H={\left(\frac{\partial \Phi \left(X,P\right)}{\partial X}\right)}^{-1}\frac{\partial \Phi \left(X,P\right)}{\partial P}$.

Suppose the uncertainties of the extracted pixel coordinates conform to the two-dimensional normal distribution with the mean value equals to zero:

(30) $$\sigma_{ui}{}^{2}=\sigma_{vi}{}^{2}=\sigma_{0}{}^{2}\left(i=1,2,\cdots ,n\right)$$

We have:

(31) $$Q_{X}=HH^{T}\sigma_{0}{}^{2}$$

where $\sigma_{0}^{2}$ is the uncertainty of the featured points introduced by the image processing error.

Equation (31) has revealed the influence rule between the measuring uncertainty of *P* and the uncertainty of *X*. As we can see, in order to decrease the uncertainty of *X*, we should not only decrease the uncertainty of *P* (the extracted image coordinates), but also decrease the influence of the uncertainty of *P*. Method to decrease the uncertainty of *P* can be using high-resolution camera or improving accuracy of image processing algorithm, and method to decease the influence of the uncertainty of *P* is to decrease the trace of *HH^T^* by optimizing the camera measuring pose.

**Figure 5:**
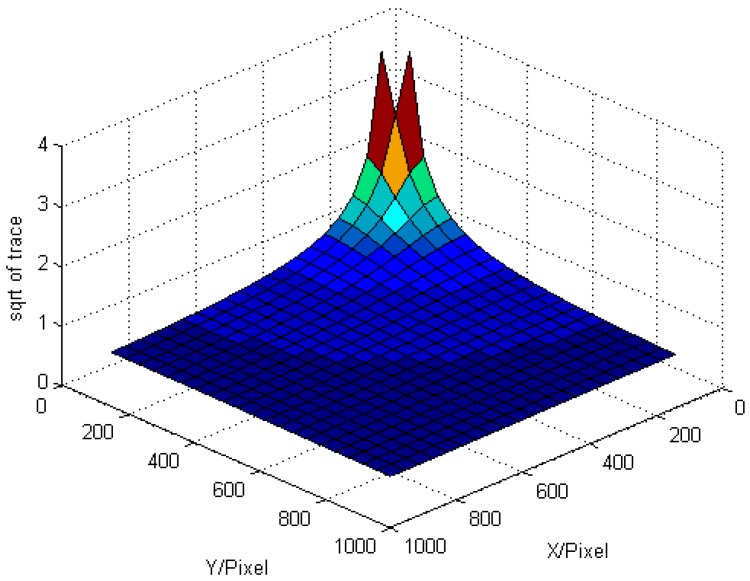
Simulation of the uncertainty.

In this paper, we take the roof intelligent grasping as an example, the camera has to move to 4 different poses and measure four different featured points on the roof. In order to obtain a clear picture of the featured points, the camera usually stops right above the featured points. That is the measuring orientations of the camera have been limited by the featured points, but the positions of the camera can be optimized. In the simulation experiment, we adjust the camera positions and make the pixel image coordinates of the featured points move from the image center to the image boundaries. The root of the trace of *HH^T^* changes as shown in Figure 5, where *X* and *Y* mean the pixel distances away from image center along in two directions. 

As we can see in Figure 5, when the featured point moves far from the principle point, the influence of the uncertainty of the image points become less, but the decrease trend becomes slower when the distance is more than 400 pixels (the simulated camera has a resolution of 2456 × 2058). When applying to the actually engineering project, in consideration of lighting conditions and other factors, we suggest that the featured points image at about 1/4 length away from the edge of the image plane.

## 5. Experiments

### 5.1. Experiment Setup

**Figure 6:**
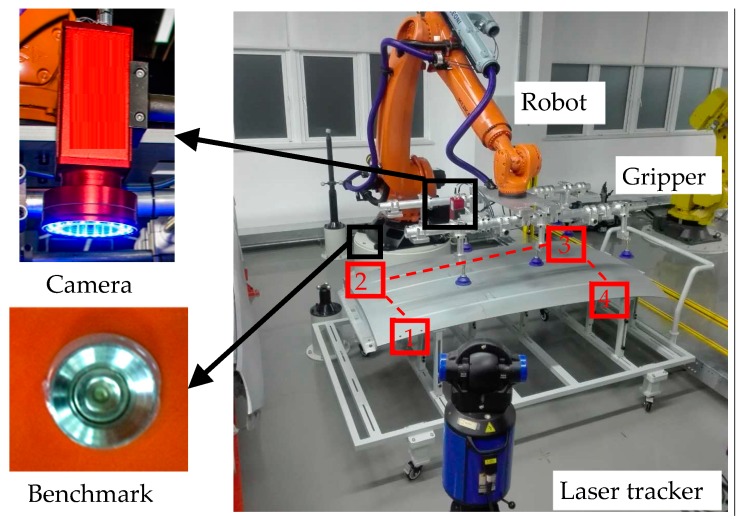
Experimental setup for robot intelligent grasping system.

In order to verify the mathematic model of the monocular 6-DOF pose estimation technology and the calibration method proposed in this paper, a robotic intelligent grasping system was setup and experimental test was performed. As shown in Figure 6, the experimental setup consists of a KR150R3100 industrial robot (KUKA, Augsburg, Germany) a PoE-B2520M-SC000 CCD camera (Imperx, Boca Raton, FL, USA) with 2/3 inch optical format and 2456 × 2058 resolution, a LM8JC10M lens (KOWA, Nagoya, Japan) with 8.5 mm focal length. The camera is mounted on the robot end, an external monochromatic light source is mounted in front of the camera. A roof part is used as the test object, which is about 2000 mm in length and 1200 mm in width. The roof is a typical large-size automobile part and the camera has to move to at least four different positions to cover it. A *Xi* laser tracker (Faro, Orlando, FL, USA) is used as high accuracy measuring equipment to calibrate the system and verify the accuracy. According to the specification, the typical measurement uncertainty (deviation between the measured and the nominal coordinate of a tested point) of the laser tracker can reach 0.025 mm within 70 m. The measuring trajectory is illustrated in Figure 6, the robot has to move to four different positions (1~4 in Figure 6) in order to measure the featured points on the roof and there are four camera locations.

### 5.2. Calibration Results

Before carrying out the experimental test, the robot intelligent grasping system should be calibrated with the method proposed in Section 2.2. As shown in Figure 6, three sophisticated magnetic holders for the SMR are fixed near the robot base, which are used as the benchmarks. CF is established firstly by measuring centers of these three SMRs. Then the roof is places at the initial pose, and the laser tracker measures four featured holes on the roof. WF is defined with these four featured holes, and the transform relationship between the WF and CF (Tw0c) is calibrated:

Tw0c=[−0.9998−0.0131−0.0120−1381.2590.0129−0.99990.0085−152.268−0.01210.00840.9999383.6420001]

Center coordinates of the featured holes on the roof under WF are given in Table 1:

Before the experimental test, the camera is calibrated with Zhang’s method [18] in advance, intrinsic parameters of camera are given in Table 2. 

The robot takes the camera to measure the roof at four different pose, and a SF is defined at each measuring pose. Transform relationship between SFi and CF are:

Ts1c=[0.9968−0.0798−0.00981131.319−0.0799−0.99680.0099−851.1020.0090−0.0107−0.9999666.9910001]Ts2c=[0.9968−0.0797−0.0093662.850−0.0798−0.99680.0101−822.7570.0084−0.0108−0.9999653.0770001]

Ts3c=[0.9968−0.0796−0.0094712.561−0.0797−0.99680.0096−149.1070.0086−0.0103−0.9999654.0120001]Ts4c=[0.9968−0.0797−0.00971157.534−0.0798−0.99680.0097−178.3390.0090−0.0105−0.9999660.3790001]

BF is established as shown in Figure 1, and the transformation from CF to BF (Tcb) is:

Tcb=[−0.9995−0.0302−0.0008325.2520.03020.9995−0.0005298.0270.00080.00050.999928.0370001]

Till now, the system calibration has finished, and pose of the roof in CF can be estimated when the robot takes the camera to measure it.

### 5.3. Image Processing Method

**Figure 7:**
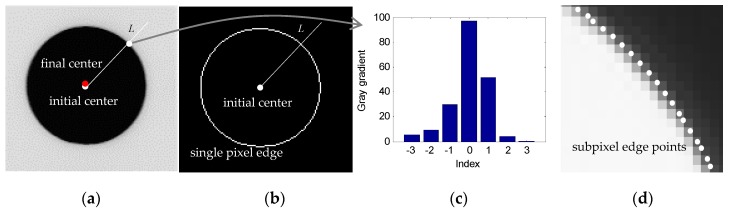
Subpixel extraction of the featured hole center: (**a**) Original image; (**b**) Single pixel edge; (**c**) Gray gradient; (**d**) Subpiexl edge points.

In this system, holes on the roof are chosen as featured points, and the picture taken by the camera is shown in Figure 7. After the camera takes picture of the featured holes, the image coordinates of the hold centers should be obtained by the image processing. Based on the perspective projection model, a spatial circle will be imaged as an ellipse on the image plane. When the camera optical axis is nearly perpendicular to the part surface, the center of the ellipse can be regarded as the projection of the hole center in image plane.

This section simply introduces the image processing method of subpixel edge extraction and ellipse fitting. First, for an original image (shown in Figure 7a), by threshold division and simple morphology, the connected domain of the ellipse is extracted and the single pixel edge of the ellipse is obtained using erosion (shown in Figure 7b). Then the initial center of the ellipse can be obtained by fitting the ellipse with the least-square method (white point in Figure 7a,b). For each pixel of the edge, along the direction of the pixel and the initial center (*L* in Figure 7a,b), the gray gradient (shown in Figure 7c) can be calculated, and the subpixel edges can be obtained by gravity method of the gradient (shown in Figure 7d). At last, the high-accuracy ellipse equation in pixel image is calculated by fitting ellipse with the least-square method again, and the final center is obtained (red point in Figure 7a).

### 5.4. Accuracy Validation Experiments

To verify the accuracy of the monocular-based 6-DOF pose estimation technology proposed in this paper, the accuracy validation experiments are carried out on the experimental setup in Figure 6. After system calibration, we put the roof in several arbitrary poses. The pose of the deviated roof relative to the initial pose was firstly measured by the laser tracker (LT). Then the robot took the camera to capture pictures of the featured holes and pose of the deviated roof was calculated with the mathematic model proposed in this paper (*Mono*). 

The 3 × 3 rotation matrix of the relative transformation relationship is described with a triple of Euler angles (*ψ*,*θ*,*φ*) here, which represent the rotation angle about *x*-axis, *y*-axis and *z*-axis respectively. The experiment was repeated five times and the comparative results (Δ = *Mono* − *LT*) are shown in Table 3, the comprehensive angle and position error are calculated as:

(32) $$\{\begin{array}{c} dR=\sqrt{\Delta \psi^{2}+\Delta \theta^{2}+\Delta \phi^{2}}\\ dT=\sqrt{\Delta x^{2}+\Delta y^{2}+\Delta z^{2}} \end{array}$$

Compared with the laser tracker measured results, the RMS (root mean squared) error for the angle and position is about 0.0228° and 0.4603 mm, which has reached the accuracy requirements of the robot intelligent grasping system.

**Figure 8:**
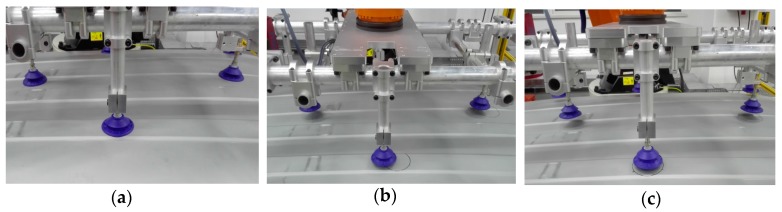
Status of the gripper: (**a**) Initial part with initial path; (**b**) Moved part with initial path; (**c**) Moved part with corrected path.

As we can see in Figure 6, the gripper for the car roof consists of a steel structure and several pneumatic chucks, and the curved surface constructed by the chucks is consistent with the roof surface. When the roof was at the initial pose, an initial robot grasping path was taught and each of the chucks fit closely to the roof surface, as shown in Figure 8a. If the roof does not deviate from the initial pose, the gripper could grasp it repeatedly along the initial path. In this experiment, we put the roof in several arbitrarily poses, and when the robot moved to grasp the roof along the initial path, chucks would deviate from the taught positions and not fit to the roof surface, as shown in Figure 8b, that is the roof cannot be absorbed. Then the part pose was determined with the method proposed in this paper and the robot grasping path was corrected based on the principle described in Section 2.1, the corrected robot grasping pose was shown in Figure 8c. Note that the chucks of the gripper was right above the absorbing positions and not fit to roof fully in Figure 8c, this is because we have stopped the robot moving to the final position in order to show a comparative effectiveness with Figure 8b.

## 6. Conclusions

A monocular 6-DOF pose estimation technology for a robot intelligent grasping system has been presented in this paper. This method is designed to be applied on the production line with large part intelligent grasping requirements and adjust the robot’s path to adapt to the pose of the deviated part. An industrial camera is mounted on the robot’s end-effector, and the robot uses the sensor to measure the featured points on the part from different poses. The mathematic model for monocular 6-DOF pose estimation technology is proposed in this paper, including the nonlinear optimization equation about the camera object space collinearity error in different poses and the estimation of the initial iteration value. According to uncertainty analysis, camera poses are optimized to reduce the influence of image processing error. Also, calibration method for the robotic system is introduced. Experimental results show that the RMS angle and position error is about 0.0228° and 0.4603 mm. It has proved that the method is feasible and valid in maintaining the accuracy of the robot intelligent grasping system. Since it is easy and convenient to implement, we expect the proposed monocular 6-DOF pose estimation technology will have wide application on the manufacturing floor for intelligent grasping. Future effort will also be devoted to extend application of this method to other robotic intelligent systems.

## Figures and Tables

**Table 1:** Coordinates of the featured points on the roof.

Point	*X*	*Y*	*Z*
*P*_*w*1_	0	0	0
*P*_*w*2_	1218.078	0	0
*P*_*w*3_	1218.028	2058.764	0
*P*_*w*4_	−1.017	2059.039	−2.677

**Table 2:** Intrinsic parameters of camera.

*a_x_*	*a_y_*	*u*_0_	*v*_0_	*k*1	*k*2	*p*1	*p*2
2421.402	2419.707	1237.997	971.382	−0.02568	0.07839	0.00103	0.0007

**Table 3:** Measured results compared with tracker.

	*ψ* (°)	*Θ* (°)	*Φ* (°)	*dR* (°)	*x* (mm)	*y* (mm)	*z* (mm)	*dT* (mm)
LT	−2.31544	3.407908	−3.50354		−31.260	−12.268	33.642	
Mono	−2.30752	3.405357	−3.47866		−31.246	−12.335	34.239	
Δ	0.00792	−0.00255	0.02488	0.026234	0.014	−0.067	0.597	0.600911
LT	−2.27619	−3.79836	−3.30576		28.382	−26.748	45.021	
Mono	−2.28559	−3.7911	−3.29375		28.285	−26.815	45.161	
Δ	−0.0094	0.007264	0.012007	0.016893	−0.097	−0.067	0.14	0.183025
LT	4.28209	2.254749	−2.33946		−39.525	52.237	−43.422	
Mono	4.296024	2.238525	−2.35284		−39.494	52.295	−44.117	
Δ	0.013934	−0.01622	−0.01338	0.025225	0.031	0.058	−0.695	0.698105
LT	−4.6618	2.774759	−4.95201		38.082	−36.264	−33.598	
Mono	−4.66049	2.761622	−4.96092		38.19	−36.41	−33.677	
Δ	0.001316	−0.01314	−0.00892	0.015932	0.108	−0.146	−0.079	0.198043
LT	4.084893	4.630798	−2.95192		−50.432	−46.475	34.246	
Mono	4.108526	4.643033	−2.95696		−50.472	−46.832	34.342	
Δ	0.023633	0.012235	−0.00504	0.027085	−0.04	−0.357	0.096	0.37184
